# Haemolymphangioma of the small bowel mesentery in adults: two case reports and a literature review

**DOI:** 10.1186/s12876-021-01855-w

**Published:** 2021-07-03

**Authors:** Yao Du, Jiang Nan Zhang, Lu Lu Zhu, Yi Wang, Wei Ping Li

**Affiliations:** 1 Department of Gastrointestinal Surgery, The First people’s Hospital of Taicang City, Taicang Affiliated Hospital of Soochow University, Taicang City, 215400 Jiangsu Province China; 2 Department of Gastrointestinal Surgery, The First Affiliated Hospital of Nanchang University, Nanchang City, 330006 Jiangxi Province China; 3 Department of Pathology, The First people’s Hospital of Taicang City, Taicang Affiliated Hospital of Soochow University, Taicang City, 215400 Jiangsu Province China

**Keywords:** Lymphangioma, Haemolymphangioma, Mesenteric tumour, Small bowel

## Abstract

**Background:**

Haemolymphangioma arising from the small bowel and its mesentery is extremely rare in the clinical setting. To date, only 8 cases of small bowel haemolymphangioma have been reported, and there have been no previously reported cases of haemolymphangioma in the small bowel mesentery (PubMed). The formation of this tumour is mostly congenital, but the exact mechanism is still unclear. As a benign tumour, the presentation of the disease may vary from a simple well-defined cystic lesion to an aggressive ill-defined lesion mimicking malignancy. However, there are no typical symptoms, and preoperative diagnosis is difficult.

**Case presentation:**

We present two cases of haemolymphangioma in the small bowel mesentery in a 54-year-old man and a 52-year-old woman. Both of them came to the hospital due to an abdominal mass. In the first case, a cystic teratoma in the left abdominal area was considered after abdominal plain computed tomography (CT) and magnetic resonance imaging (MRI) scans. After taking an enhanced CT scan, a lipoma was considered based on the images. In the second case, cystic masses of the left upper and middle abdomen were observed on abdominal ultrasonography. An abdominal plain CT scan showed an irregular low-density mass in the left upper and middle abdomen. With an enhanced CT scan, haemolymphangioma was considered based on the images. After complete surgical removal, the masses were found to originate from the small bowel mesentery and had not invaded into the peripheral lymphatic tissue. In case 1 in this study, the routine pathology diagnosis was lymphangioma, while in case 2, the diagnosis was haemangioma. The final diagnosis was confirmed to be haemolymphangioma by immunohistochemistry in both cases. No recurrence was evident during 4 months of follow-up. We review the previous case reports of haemolymphangioma in the abdominal cavity and discuss their clinical features, diagnosis, treatment and prognosis.

**Conclusions:**

The clinical manifestations of abdominal haemolymphangiomas can vary for both location and size. Abdominal CT examination has important clinical value for haemolymphangioma in the abdominal cavity. The final diagnosis of haemolymphangioma depends on a postoperative pathological examination. In addition, postoperative regular follow-up is necessary.

## Background

Haemolymphangioma (also called vascular tumour) is a rare type of lymphangioma that shows a mixture of blood vessels and lymphatics. Haemolymphangioma most commonly presents in infants and young children on the body surface. The incidence of haemolymphangioma in adults is low, and there are only a few reports in the literature. In adults, most haemolymphangiomas occur in the head and neck, while only a few cases have been reported to occur in the digestive system, such as the liver [1], pancreas [2, 3], duodenum [4, 5], small bowel [6–11] and rectum [12]. No case of haemolymphangioma has been reported in the small bowel mesentery before (PubMed). Here, we report the first two cases of haemolymphangioma of the small bowel mesentery in a 54-year-old man and a 52-year-old woman. The clinical features, diagnosis, treatment and prognosis of haemolymphangioma cases reported in the literature were reviewed.

## Case presentation

### Patient no. 1

**Fig. 1:**
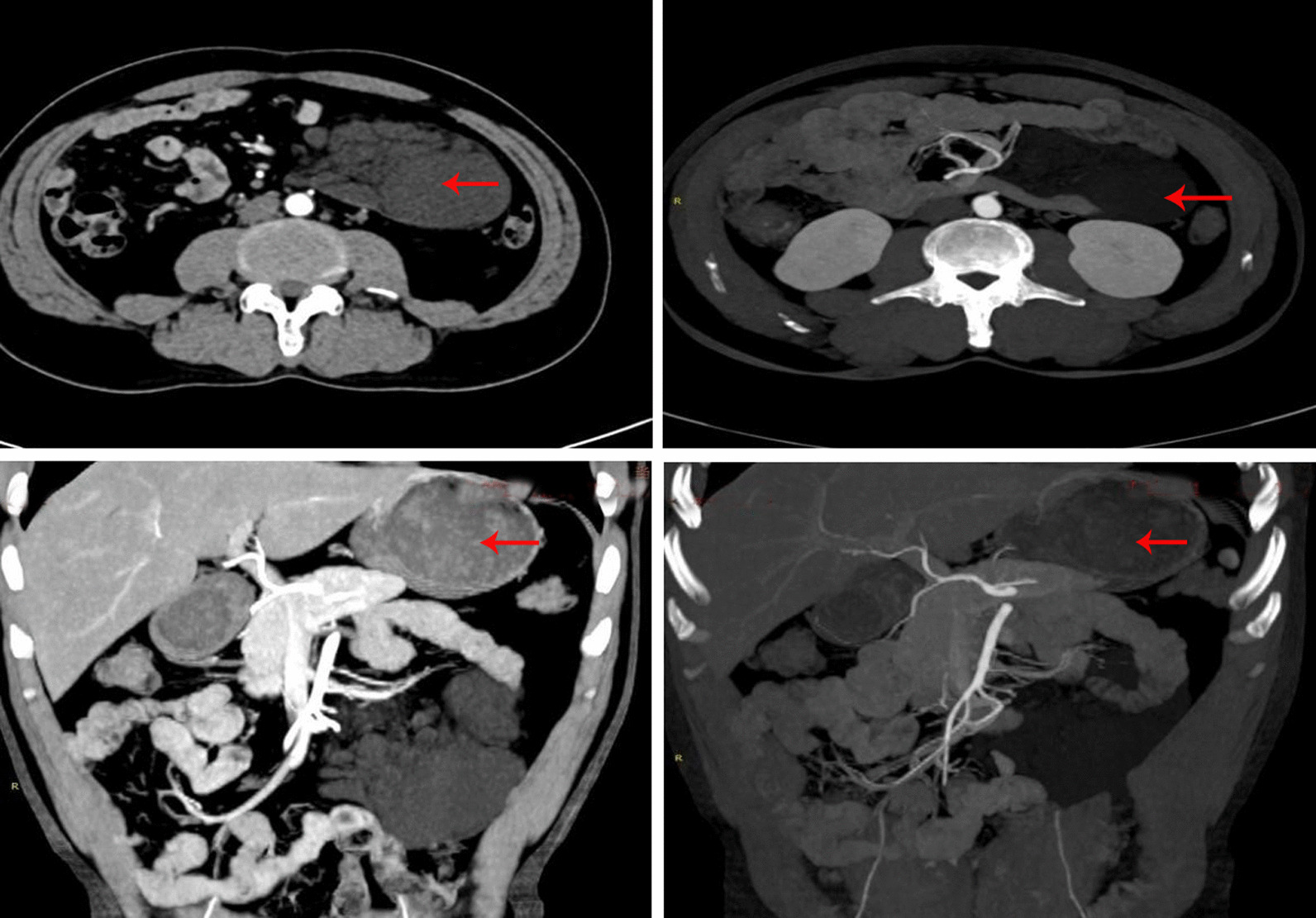
Abdominal enhancement CT revealing an irregular mass (arrows) originating from the mesentery in the left mid-abdomen. The maximum cross-sectional area of the mass was approximately 5.5 cm × 10.6 cm, and a low-density shadow of the fat inside can be observed. The contrast-enhanced CT scans show no obvious enhancement. The mass constricted the adjacent small bowel and pushed it out (Case 1)

A 54-year-old Chinese man came to the hospital due to an abdominal mass he had noticed 4 days prior. Abdominal plain CT and MRI scans showed an abdominal cystic mass that was considered to be a cystic teratoma. The patient had a history of inguinal hernia surgery. An abdominal examination showed no positive signs. Routine blood, blood biochemistry, tumour biomarkers (AFP, CEA, CA12-5, and CA199), other blood tests and urine analysis were normal. Abdominal enhanced CT revealed (Fig. 1) an irregular mass originating from the mesentery in the left mid-abdomen, and the maximum cross-sectional area of the mass was approximately 5.5 cm × 10.6 cm. Contrast-enhanced CT scans showed no obvious enhancement of the lesion. Radiologically, it was diagnosed as possibly a lipoma.

**Fig. 2:**
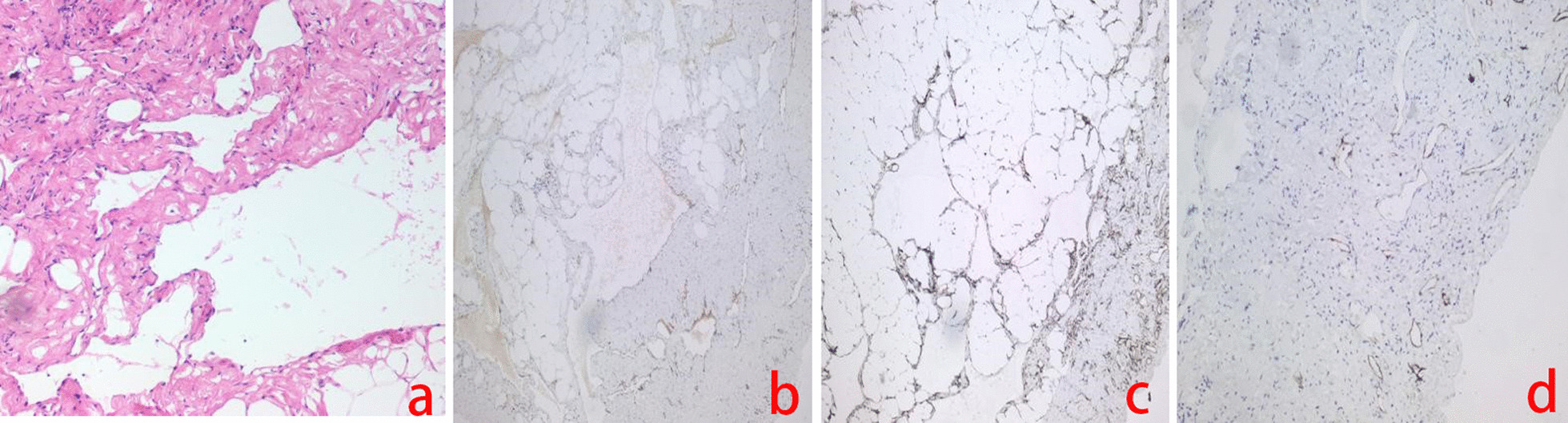
Pathological examination in Case 1. Haematoxylin-eosin staining showing some lymphatic vessels (**a**) (×100). Immunohistochemical staining is positive for CD31 (**b**) and CD34 (**c**) in the vascular endothelial cells and positive for D2-40 (**d**) staining in the lymphatic endothelial cells (×100)

The patient underwent laparoscopic exploration and surgical resection. During surgery, a cystic lesion of 8 cm × 10 cm originating from the small bowel mesentery was observed in the jejunum approximately 20 cm distal to the ligament of Treitz. The tumour was completely resected. A lipoma was diagnosed by rapid intraoperative pathology. Postoperative histological examination revealed that the mass had lumens of varying sizes with lymphatic fluid and lymphocytes, which prompted a final pathological diagnosis of lymphangioma. Further immunohistochemical staining showed CD31 (blood vessel +), CD34 (blood vessel +), and D2-40 (lymphatic vessel +) positive reactions (Fig. 2a–d). Finally, the diagnosis was confirmed to be haemolymphangioma originating from the small bowel mesentery. The patient recovered well and was discharged 8 days after surgery. No tumour recurrence was observed after 4 months of follow-up.

## Patient no.2

**Fig. 3:**
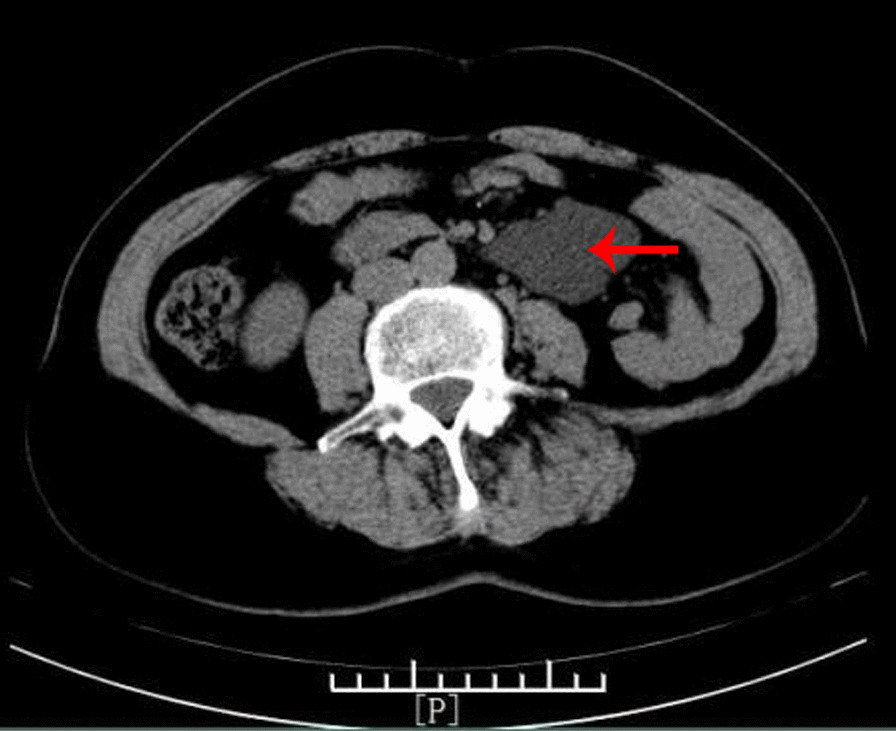
Plain abdominal CT scan showing an abdominally irregular low-density mass of 5.9 cm × 4.1 cm on October 25, 2020 (Case 2)

**Fig. 4:**
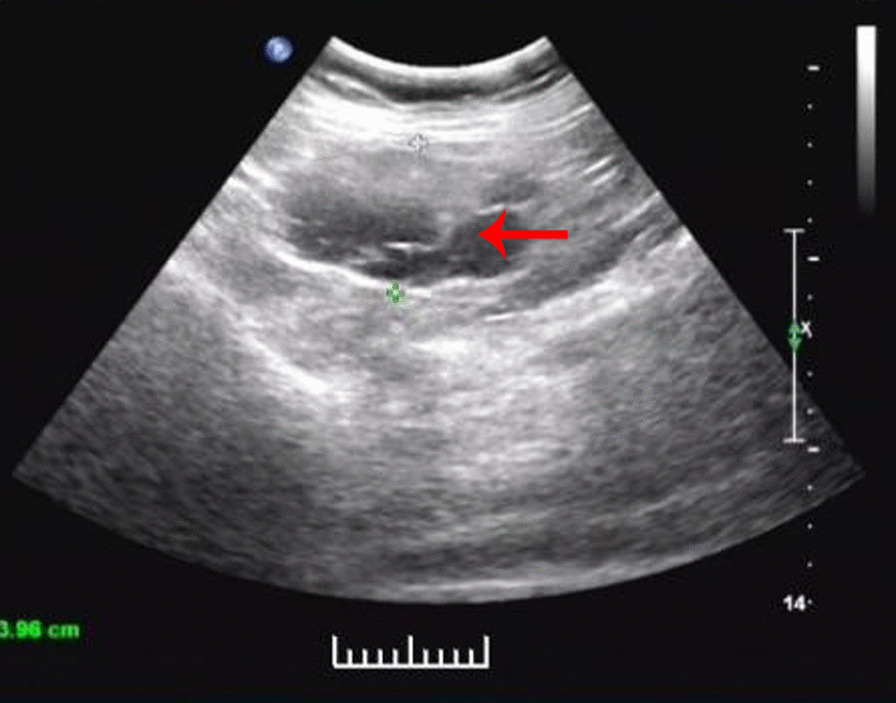
Abdominal ultrasonography showing an abdominal cystic mass of 6.9 cm × 4.0 cm in the left upper and middle abdomen, and colour Doppler imaging showing a spotty colour blood flow signal around the lesion (Case 2)

**Fig. 5:**
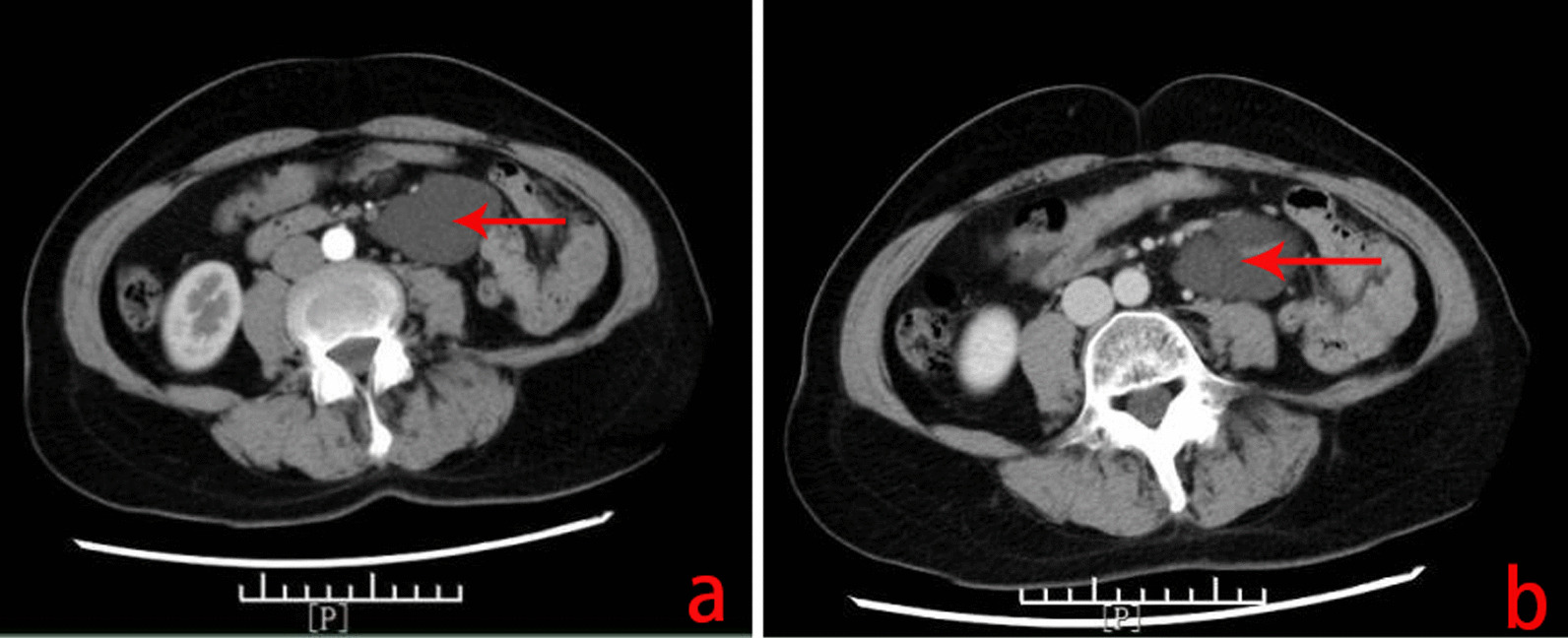
Enhanced CT scan showing an abdominal low-density mass (arrows) of 5.9 cm × 4.1 cm. The CT value was approximately − 2 to − 4 HU, and there was no significant enhancement in the arterial phase (**a**) but slight enhancement in the venous phase (**b**) (Case 2)

A 52-year-old Chinese woman came to the hospital on November 2, 2020 due to an abdominal mass she had noticed 3 years prior. Her prehospital abdominal plain CT scan showed an abdominal irregular low-density mass of 4.5 cm × 2.5 cm on March 14, 2020. A review of a plain abdominal CT scan on October 25, 2020 (Fig. 3) showed that the abdominal mass was slightly larger than before (5.9 cm × 4.1 cm). The patient had a history of laparoscopic total hysterectomy and bilateral salpingectomy for 3 years. Abdominal examination showed no positive signs. Routine blood, blood biochemistry, tumour biomarkers (AFP, CA199, and CA12-5), other blood tests and urine analysis were normal. On abdominal ultrasonography (Fig. 4), cystic masses of 6.9 cm × 4.0 cm were observed in the left upper and middle abdomen. An enhanced CT scan (Fig. 5a, b) showed that the abdominal cystic focus was similar in size (5.9 cm × 4.1 cm) to the mass noted on October 25, 2020. There was no significant enhancement in the arterial phase but slight enhancement in the venous phase, and its diagnosis was considered to be haemolymphangioma.

**Fig. 6:**
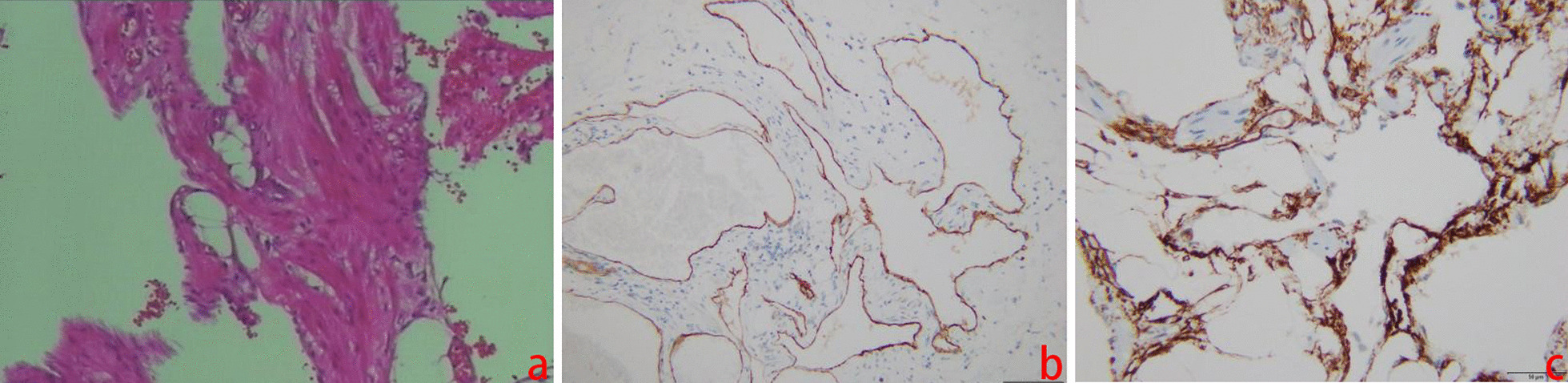
Pathological examination in Case 2. Haematoxylin-eosin staining showing some blood vessels (×100) (**a**). Immunohistochemical staining showing positivity for CD34 in vascular endothelial cells (×100) (**b**). Immunohistochemical staining showing positive D2-40 staining in lymphatic endothelial cells (×100) (**c**)

The patient underwent laparoscopic resection of the tumour. During surgery, a cystic lesion of 5 cm × 5 cm originating from the small bowel mesentery was observed in the jejunum approximately 15 cm distal to the ligament of Treitz. The mass was completely resected. Postoperative histopathological examination revealed that the tumour had lumens of varying sizes with red blood cells, with a pathological diagnosis of haemangioma. Immunohistochemical staining showed CD34 (blood vessel +) and D2-40 (lymphatic vessel +) positive reactions (Fig. 6a–c). The final diagnosis was haemolymphangioma of the small bowel mesentery. The patient recovered well and was discharged 4 days after surgery. No tumour recurrence was observed after 4 months of follow-up.

## Discussion and conclusions

Lymphangiomas are a heterogeneous group of vascular malformations that are comprised of dilated lymphatic spaces. They are benign malformations and are classified into four categories [13]: capillary lymphangioma, cavernous lymphangioma, cystic lymphangioma (hygroma) and haemolymphangioma (a combination of haemangioma and lymphangioma). Its incidence varies from 1.2 to 2.8 per 1000 newborns, and both sexes are equally affected [6]. Although lymphangiomas can occur at any age and in any part of the body, approximately 90 % of cases occur in children who are less than 2 years of age, most commonly on the head and neck. These lesions are rarely found in adult patients [6].

Haemolymphangioma arising from the small bowel and its mesentery is extremely rare in the clinic. We reviewed the literature by searching for “haemolymphangioma [All Fields] OR haemangiolymphangioma [All Fields]” in PubMed. There have been only 8 cases reported on small bowel haemolymphangioma (Table 1) [4–11] and there are no prior reports of haemolymphangioma in the small bowel mesentery. The formation of this tumour is mostly congenital, but the exact mechanism is still unclear. Some researchers have considered it to be a congenital malformation of the lymphatic vascular system, while others considered it to be induced by poor lymph drainage and lymphatic damage as a result of trauma or surgery [14, 15].

**Table 1:** The 8 cases previously reported of small bowel haemolymphangioma

Case No.	References	Year	Age (years)	Sex	Clinical symptoms	Location	Tumour size (cm)	Diagnostic method	Treatment
1	Fang et al. [6]	2012	57	F	Melena, anaemia	30 cm distal to Treitz	5.0	Enteroscopy	Surgical resection
2	Antonino et al. [4]	2014	24	F	Anaemia	Second portion of duodenum	5.0	Gastroduodenoscopy	Surgical resection
3	Gómez-Galán et al. [5]	2016	43	F	Chronic anaemia	Distal duodenum	4.0	Capsule endoscopy and enteroscopy	Surgical resection
4	Blanco et al. [7]	2017	45	F	Melena, anaemia	90 cm distal to Treitz	8.0	Capsule endoscopy and enteroscopy	Laparoscopic small bowel resection
5	Iwaya et al. [8]	2018	70	M	Melena, anaemia	120 cm distal to Treitz	2.0	Capsule endoscopy and enteroscopy	Laparoscopic small bowel resection
6	Yang et al. [9]	2019	20	F	Melena, anaemia	60 cm distal to Treitz	10.0	Computed tomography scan and enteroscopy	Laparoscopic small bowel resection
7	Xiao et al. [10]	2020	42	M	Melena, anaemia	150 cm distal to Treitz	-	Capsule endoscopy and enteroscopy	Enteroscopic injection sclerotherapy
8	Teng et al. [11]	2020	55	F	Abdominal discomfort	60 cm distal to Treitz	3.0	Computed tomography scan and enteroscopy	Surgical resection

### Case 2

in this study had undergone laparoscopic total hysterectomy and bilateral salpingectomy three years prior. Surgical injury to her blood vessels and lymphatics has not been ruled out, and it is also possible that the previous surgical exploration missed the haemolymphangioma. In Case 1 in this study, inguinal hernia surgery was performed as an open procedure and did not penetrate the abdominal cavity. Therefore, it could not affect the small bowel mesenteric vascular system.

The clinical manifestations of abdominal haemolymphangiomas can vary in both location and size. Abdominal pain and an abdominal mass are common symptoms in patients with haemolymphangioma of the pancreas [2, 3]. Haemolymphangioma of the digestive tract is characterized by anaemia and gastrointestinal bleeding [4, 6, 9, 12]. Small intra-abdominal cysts may not present any symptoms unless they enlarge significantly and constrict adjacent organs. Pandey et al. [16] reported a case of a large haemolymphangioma of the greater omentum in a 3-year-old boy who was hospitalized with intractable abdominal pain. The clinical manifestation of our two cases was an abdominal mass without other symptoms. However, tumours of the small bowel mesentery can cause abdominal pain, bowel obstruction, and gastrointestinal bleeding when they constrict the adjacent small bowel or rupture.

Due to the difficulty in distinguishing haemolymphangioma from other small bowel diseases, a preoperative diagnosis is extremely hard. Routine blood tests and tumour biomarkers (such as AFP, CEA, CA199, and CA12-5) usually show normal results. Gastrointestinal haemolymphangioma is clinically diagnosed mainly based on endoscopy, such as tumours located in the duodenum [4, 5], small bowel [6–11] and rectum [12]. The clinical diagnosis of nongastrointestinal haemolymphangioma mainly depends on ultrasonography, CT and MRI, such as tumours located in the liver [1], pancreas [2] and greater omentum [16]. Abdominal CT examination has important clinical value for diagnosing haemolymphangioma in the abdominal cavity. The different proportions of blood vessels in haemolymphangioma may lead to different enhanced characteristics upon imaging. Significant enhancement is usually observed in tumours rich in blood vessels, and the enhancement is more obvious in the venous phase and the delayed phase. The enhancement is not obvious in a tumours with a low proportion of blood vessels, leading to easy misdiagnosis. In addition, CT is useful in defining the extent and invasion of the mass and for planning the surgical strategy [17]. Typically, MRI can also assist in determining the association of haemolymphangioma with the surrounding tissues, as well as the extent of invasion [18].

### Case 1

in our study underwent a contrast-enhanced CT scan of the abdomen and showed no significant enhancement due to the low proportion of blood vessels in the tumour. In Case 2, the enhancement was not significant in the arterial phase during contrast-enhanced scanning; however, enhancement was seen in the venous phase. Radiologically, it was diagnosed as possibly haemolymphangioma.

The final diagnosis of haemolymphangioma depends on postoperative pathological examination. The architecture of the constituent vessels on routine haematoxylin-eosin (H&E) histological preparations is usually distinguished. Further identification can be made by immunohistochemistry in such cases. Both vascular endothelial cells and lymphatic endothelial cells are CD31- and CD34- positive, while D2-40 is only expressed in lymphangioma and some malignant vascular tumours. Therefore, immunoreactivity for podoplanin with the D2-40 antibody helps to differentiate it from lymphatic endothelial cells [19]. Combined with the abovementioned indicators, it is not difficult to diagnose haemolymphangioma. In Case 1 in this study, the routine pathology diagnosis was lymphangioma, while in Case 2 in this study, the routine pathology diagnosis was haemangioma. CD34 (blood vessel+) and D2-40 (lymphatic vessel+) were observed by immunohistochemistry in two cases, which further confirmed the final diagnosis of haemolymphangioma.

Abdominal haemolymphangiomas are benign lesions, but the tumour growth may be rapid. It can compress the intestinal canal and surrounding parenchymal organs to cause abdominal pain, abdominal distension, bowel obstruction and other acute abdominal symptoms, and the tumour itself may bleed, rupture, or infect. Therefore, once the above signs occur, the patients should be treated in a timely manner. The standard management of haemolymphangioma is surgical resection. Other nonsurgical treatments, including cryotherapy, laser therapy, radiotherapy and local injection of sclerotic agents, compared with surgical treatment, do not show superiority [13]. It has been reported in the literature that lesions after complete surgical resection have a 10–27 % rate of recurrence, while 50–100 % of partly resected tumours may recur [6]. Therefore, regular postoperative follow-up is necessary. Our two patients were in a good state after surgery, and no complications occurred. There were no signs of tumour recurrence at 4 months after the operation.

Two cases of haemolymphangioma of the small bowel mesentery were reported here. The clinical features, diagnosis, treatment and prognosis of haemolymphangioma were reviewed in the literature. However, haemolymphangioma of the small bowel mesentery is still poorly understood because it rarely occurs in adults. Clinicians need to improve their awareness of haemolymphangioma.

## Data Availability

All data and materials are presented in this manuscript.

## Abbreviations

CT: Computed tomography
MRI: Magnetic resonance imaging
CD: Cluster of differentiation
H&E: Haematoxylin-eosin

## References

[1] Hu HJ, Jing QY, Li FY (2017). Hepatic hemolymphangioma manifesting as severe anemia. J Gastrointest Surg.

[2] Sun LF, Ye HL, Zhou QY (2009). A giant hemolymphangioma of the pancreas in a 20-year-old girl: a report of one case and review of the literature. World J Surg Oncol.

[3] Toyoki Y, Hakamada K, Narumi S (2008). A case of invasive hemolymphangioma of the pancreas. World J Gastroenterol.

[4] Antonino A, Gragnano E, Sangiuliano N (2014). A very rare case of duodenal hemolymphangioma presenting with iron deficiency anemia. Int J Surg Case Rep.

[5] Gómez-Galán S, Mosquera-Paz MS, Ceballos J (2016). Duodenal hemangiolymphangioma presenting as chronic anemia: a case report. BMC Res Notes.

[6] Fang YF, Qiu LF, Ying D (2012). Small intestinal hemolymphangioma with bleeding: a case report. World J Gastroenterol.

[7] Blanco Velasco G, Tun Abraham A, Hernández Mondragón O (2017). Hemolymphangioma as a cause of overt obscure gastrointestinal bleeding: a case report. Rev Esp Enferm Dig.

[8] Iwaya Y, Streutker CJ, Coneys JG (2018). Hemangiolymphangioma of the small bowel: a rare cause of chronic anemia. Dig Liver Dis.

[9] Yang JZ, Zhang Y, Kou GJ (2020). Jejunum hemolymphangioma
causing refractory anemia in a young woman. Am J Gastroenterol.

[10] Xiao NJ, Ning SB, Li T (2020). Small intestinal hemolymphangioma treated with enteroscopic injection sclerotherapy: a case report and review of literature. World J Gastroenterol.

[11] Teng YJ, Wang J, Xi QH (2020). Jejunal hemolymphangioma: a case report. Medicine.

[12] Chen G, Cui W, Ji XQ (2013). Diffuse hemolymphangioma of the rectum: a report of a rare case. World J Gastroenterol.

[13] Kosmidis I, Vlachou M, Koutroufinis A (2010). Hemolymphangioma of the lower extremities in children: two case reports. J Orthop Surg Res.

[14] Zhang X, Sheng X, Liu F (2012). Hemolymphangioma of the chest wall: a rare case report. Oncol Lett.

[15] Li Y, Pang X, Yang H (2015). Hemolymphangioma of the waist: a case report and review of the literature. Oncol Lett.

[16] Pandey S, Fan M, Zhu J (2016). Hemolymphangioma of greater omentum: a rare case report. Medicine.

[17] Liang P, Gao J, Javier P (2015). CT findings and clinical features of pancreatic hemolymphangioma: a case report and review of the literature. Medicine.

[18] Mao CP, Jin YF, Yang QX (2018). Radiographic fifindings of hemolymphangioma in four patients: a case report. Oncol Lett.

[19] Kalof AN, Cooper K (2009). D2-40 immunohistochemistry-so far!. Adv Anat Pathol.

